# A marine-derived sesterterpenoid inhibits migration and invasion of triple-negative breast cancer cells by suppressing the FAK signaling pathway

**DOI:** 10.3389/fcell.2026.1967923

**Published:** 2026-10-15

**Authors:** Haiyan Wen, Feifei Zeng, Yu Lan, Yuping Yin, Yuxiang Huang, Yutong Hu, Jiaheng Yin, Juanjuan Li, Siqi Zhou, Kui Hong

**Affiliations:** 1 Department of Pharmacy, Renmin Hospital of Wuhan University, Wuhan, China; 2 Department of Radiology, Renmin Hospital of Wuhan University, Wuhan, China; 3 Key Laboratory of Combinatorial Biosynthesis and Drug Discovery, Ministry of Education, School of Pharmaceutical Sciences, Wuhan University, Wuhan, China; 4 Department of Breast and Thyroid Surgery, Renmin Hospital of Wuhan University, Wuhan, China; 5 Department of Orthopedics, Renmin Hospital of Wuhan University, Wuhan, China

**Keywords:** cell migration and invasion, FAK signaling pathway, MHO7, sesterterpenoid, triple-negative breast cancer

## Abstract

**Background:**

The high incidence of distant metastasis in triple-negative breast cancer (TNBC) remains the principal driver of disease-related mortality, highlighting an urgent clinical need for novel therapeutics to restrict tumor progression and dissemination.

**Methods:**

We investigated the anti-tumor potential and underlying molecular mechanisms of MHO7 (6-epi-ophiobolin G), a structurally unique sesterterpenoid derived from a marine fungus, using MDA-MB-231 cells *in vitro* and a subcutaneous xenograft model *in vivo*. Transcriptomic profiling and biochemical analyses were performed to elucidate the underlying signaling alterations.

**Results:**

Both *in vitro* and *in vivo* evaluations demonstrated that MHO7 profoundly curtails MDA-MB-231 cell migration and invasion, strengthens adherens junctions, and exhibits robust primary tumor growth inhibition without inducing overt systemic toxicity in preclinical mouse models. Mechanistically, transcriptomic profiling coupled with biochemical analyses revealed that MHO7 exerts inhibitory effects on cell migration and invasion primarily by attenuating the Focal Adhesion Kinase (FAK) signaling pathway. Specifically, MHO7 treatment remarkably suppressed the autophosphorylation of FAK (Y397) and the subsequent recruitment of p-Src (Y419). This pathway suppression consequently altered cytoskeletal architecture, attenuated the downstream PI3K/AKT signaling cascade, and curtailed tumor angiogenesis. Furthermore, we identified a dual inhibitory mechanism wherein MHO7 successfully dismantled the oncogenic crosstalk between FAK and the canonical Wnt/β-catenin pathway by significantly downregulating nuclear β-catenin accumulation.

**Conclusion:**

MHO7 exerts tumor-suppressive and anti-migratory effects in TNBC primarily by attenuating FAK signaling and its downstream pathways, supporting its potential as a promising therapeutic candidate for highly aggressive TNBC.

## Graphical Abstract

**Figure:**
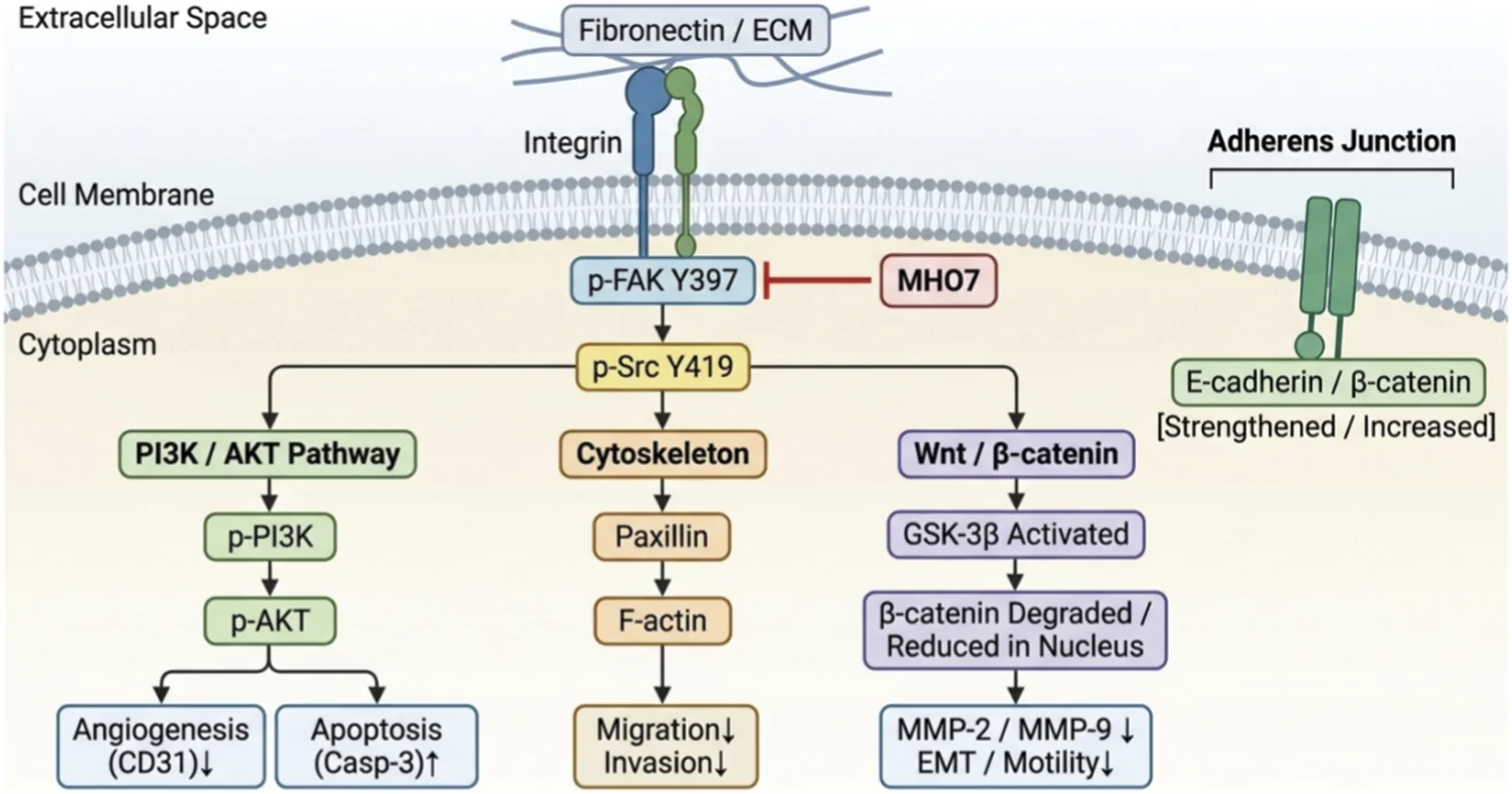

## 1 Introduction

Breast cancer remains the most commonly diagnosed malignancy among women worldwide, with both its incidence and mortality rates continuing to rise (Bray et al., 2024). Among its molecular subtypes, triple-negative breast cancer (TNBC) represents the most aggressive variant and poses a substantial clinical challenge, characterized by high intrinsic heterogeneity and a strong propensity for early metastasis, which collectively contribute to its poor clinical outcomes (Zagami and Carey, 2022). Due to the absence of well-established molecular targets, chemotherapy remains the primary therapeutic option for TNBC; however, its efficacy is often limited by the development of drug resistance and insufficient improvements in long-term survival, particularly in patients with metastatic disease (Bianchini et al., 2022). Even the emergence of novel therapeutics, such as immune checkpoint inhibitors and antibody-drug conjugates, has failed to broadly impact patient outcomes, and their efficacy is restricted to narrow, biomarker-defined populations, leaving the vast majority of patients, especially those with metastatic TNBC without effective treatment options (Zou et al., 2020; Tarantino et al., 2022). Therefore, a critical unmet need persists for novel therapeutic strategies that can effectively combat TNBC progression.

Tumor cell migration and invasion, the critical initial steps of the metastatic cascade, are the principal drivers of recurrence and cancer-related mortality in breast cancer patients (Saha et al., 2021). Central to these processes is focal adhesion kinase (FAK), a non-receptor tyrosine kinase that acts as a critical node in cell-extracellular matrix interactions and integrin-mediated signaling, thereby governing cell survival, motility and invasion (Chuang et al., 2022). Accumulating evidence reveals that FAK is frequently overexpressed and hyperactivated in multiple malignancies, and is strongly correlated with poor prognosis (Chen et al., 2025; Han et al., 2025). In TNBC, aberrant FAK activation orchestrates malignant progression, driving invasion and chemoresistance, serving as a robust predictor of adverse clinical outcomes (Rigiracciolo et al., 2019; Murphy et al., 2020; Fatherree et al., 2022). Furthermore, FAK has been shown to promote TNBC angiogenesis by upregulating vascular endothelial growth factor receptor 2 (VEGFR2) expression (Shiau et al., 2021). Given its indispensable role in driving TNBC progression, pharmacologically attenuating the FAK signaling pathway to restrict tumor cell motility represents a highly promising and viable therapeutic strategy.

Historically, marine environments have served as a prolific source of structurally diverse and pharmacologically active natural products. Ophiobolins, a unique class of fungal-derived sesterterpenoids, have garnered significant attention due to their potent anti-tumor properties (Gowans et al., 2024; Carbone et al., 2026). Among these, MHO7 (6-epi-ophiobolin G) is a secondary metabolite isolated from a marine fungus, which exhibits potent antitumor activity, as demonstrated in our previous studies (Wen et al., 2022; Ding et al., 2024; Wang et al., 2025). Although our recent work established that MHO7 effectively inhibits TNBC tumor growth by triggering tumor immunogenicity (Wen et al., 2022), its potential to impede malignant cell motility, specifically cell migration and invasion, remains unexplored, and the molecular mechanisms underlying such potential antitumor effects are currently unknown.

In the present study, we aimed to investigate the anti-tumor and anti-migratory effects of MHO7 on TNBC cells and to explore the associated signaling changes. Using the highly invasive mesenchymal-like TNBC cell line MDA-MB-231 as a model, we found that MHO7 significantly suppressed TNBC cell motility, accompanied by a profound attenuation of FAK phosphorylation and its downstream signaling cascades. Furthermore, *in vivo* experiments validated that MHO7 effectively suppressed TNBC subcutaneous tumor growth without significant systemic toxicity. These findings highlight the potential of the marine-derived sesterterpenoid MHO7 as a novel therapeutic agent for restricting TNBC progression.

## 2 Materials and methods

### 2.1 Cell culture and reagents

Human TNBC cell line MDA-MB-231 was purchased from the China Center for Type Culture Collection (CCTCC) (Wuhan, China), authenticated by short tandem repeat (STR) profiling, and routinely tested for *mycoplasma* contamination. Cells were cultured at 37 °C in a humidified 5% CO_2_ atmosphere containing and maintained in Dulbecco’s Modified Eagle Medium (DMEM, Gibco) supplemented with 10% fetal bovine serum (FBS, ExcellBio, Cat. #FCS500) and 1% penicillin-streptomycin (Bioss, Cat. #C7072).

As our previously reported (Chai et al., 2016), the sesquiterpene compound MHO7 (purity≥98% by HPLC) was isolated from the secondary metabolites of a marine-derived fungus. For *in vitro* experiment, MHO7 was prepared as a 10 mM stock solution in dimethyl sulfoxide (DMSO, Abcam, Cat. #FCS500) and diluted to the desired working concentration with complete culture medium before use, ensuring that the final DMSO concentration did not exceed 0.1% (v/v). Doxorubicin hydrochloride (ADR) (M12008) was purchased from Meryer Chemical Technology Co., Ltd. (Shanghai, China). Phalloidin peptide (Cat. #P5282) was obtained from Merck KGaA (Darmstadt, Germany). The primary antibodies used in this study were listed as follow: E-cadherin (Cat. #98123-1-RR, Proteintech Goup, Inc.), β-actin (Cat. # 66009-1-Ig, Proteintech Goup, Inc.), β- Catenin (Cat. # 51067-2-AP, Proteintech Goup, Inc.), GAPDH (Cat. #775172ES50, Yeasen Biotechnology Co., Ltd.), GSK3-β (Cat. #E-AB-22130, Elabscience Biotechnology Co.,Ltd.), phospho-pan-AKT1/2/3 (Ser473) (Cat. #AF3263, Affinity Biosciences), pan-AKT1/2/3 (Cat. #AF6261, Affinity Biosciences), Tubulin beta (Cat. #AF7011, Affinity Biosciences), PI3K p85 alpha (Cat. #AF6241, Affinity Biosciences), FAK (Cat. #AF6397, Affinity Biosciences), phospho-FAK (Tyr397) (Cat. #AF3398, Affinity Biosciences), Src (Cat. #AF6161, Affinity Biosciences), and phospho-Src (Tyr419) (Cat. #AF3162, Affinity Biosciences) antibodies. The secondary antibodies HRP-conjugated Goat Anti-Rabbit IgG (H + L) (Cat. #SA00001-2) and HRP conjugated Goat Anti-Mouse IgG (H + L) (Cat. #GB23301) were purchased from Proteintech Goup, Inc. and Servicebio Technology Co., Ltd.

### 2.2 Transwell assay

Transwell assay assessed cell migration and invasion. MHO7 or DMSO-treated cells were seeded into the upper chamber of Transwell inserts (Cat. #3422, Corning). For invasion, inserts were pre-coated with Matrigel (BD Biosciences). The lower chamber contained medium with 10% FBS as chemoattractant. After 24 h, non-migrated/non-invaded cells were removed from the upper surface. Then, cells on the lower surface were fixed with 4% paraformaldehyde, stained with 0.1% crystal violet, and counted across five random fields per insert.

### 2.3 Wound healing assay

Wound healing assay evaluated cell migration. Cells were seeded in 6-well plates to confluence and treated with MHO7 or DMSO for 24 h. After that, cells were scratched with a sterile 200-μL pipette tip, and washed with PBS to remove debris. Serum-free medium was added to suppress proliferation. Images of each scratch were captured at 0 h and 24 h using an inverted microscope. Wound closure was quantified in ImageJ as the percentage of initial wound area.

### 2.4 Cell adhesion detection

Cell adhesion was quantified by seeding MHO7 or DMSO-treated cells onto fibronectin-coated 96-well plates. After 30 min at 37 °C, non-adherent cells were washed away with PBS. Adherent cells were fixed, stained with 0.1% crystal violet, and measured at 570 nm.

### 2.5 Total RNA extraction and qRT-PCR

According to the manufacturer’s instructions, total RNA was extracted from cultured cells or tumor tissue using RNAsimple Total RNA Kit (Cat. #DP419, TIANGEN BIOTECH (BEIJING) CO., LTD.). RNA purity and concentration were determined by spectrophotometry (A260/A280) using Nanodrop. cDNA was synthesized from 1 μg of total RNA using the RevertAid First Strand cDNA Synthesis Kit (Thermo Scientific). qRT-PCR was performed using TransStart® Tip Green qPCR SuperMix (TransGen Biotech). Relative gene expression was normalized to GAPDH and calculated by the 2^-ΔΔCt^ method. Primer sequences are listed as below: CDH1 (F: CGG​GAA​TGC​AGT​TGA​GGA​TC, R: AGG​ATG​GTG​TAA​GCG​ATG​GC); CTNNB1 (F: GAA​ACG​GCT​TTC​AGT​TGA​GC, R: CTG​GCC​ATA​TCC​ACC​AGA​GT); MMP-2 (F: CTT​CCA​AGT​CTG​GAG​CGA​TGT, R: TAC​CGT​CAA​AGG​GGT​ATC​CAT); MMP-9 (F: GGG​ACG​CAG​ACA​TCG​TCA​TC, R: TCG​TCA​TCG​TCG​AAA​TGG​GC); GAPDH (F: GAA​AGC​CTG​CCG​GTG​ACT​AA, R: GCC​CAA​TAC​GAC​CAA​ATC​AG AG).

### 2.6 Immunofluorescence

MHO7 or DMSO-treated cells were fixed with 4% paraformaldehyde, permeabilized with 0.1% Triton X-100, and blocked with 5% BSA. Primary antibody was incubated overnight at 4 °C, followed by Alexa Fluor-conjugated secondary antibody for 1 h at room temperature. Nuclei were counterstained with DAPI. Images were acquired using a fluorescence microscope (Olympys).

### 2.7 Western blot analysis

Western blotting was used to quantify protein expression in tissue homogenates. Proteins were extracted with RIPA buffer (Beyotime) plus protease and phosphatase inhibitors (Servicebio). Concentrations were measured by BCA assay (Servicebio). Equal amounts were separated on 10% SDS-PAGE gels and transferred to PVDF membranes (Millipore). Membranes were blocked with 5% non-fat milk in TBST for 1 h at room temperature, then incubated overnight at 4 °C with primary antibodies. After TBST washes, HRP-conjugated secondary antibodies were applied for 1 h at room temperature. Bands were detected using ECL substrate (Sangon Biotech) and imaged on a ChemiDoc XRS + system (BioRad). Densitometry was performed in ImageJ and protein levels were normalized to β-actin or GAPDH.

### 2.8 Animal experiment

Female BALB/c nude mice (5 weeks old, 16–18 g) were purchased from the Animal Experiment Center of Wuhan University (ABSL-III certified facility) and housed under specific pathogen-free conditions with a 12-h light/dark cycle. To establish subcutaneous TNBC xenograft models, MDA-MB-231 cells (1–5x10^6^cell/mL) were suspended in ice-cold Matrigel (high-concentration, Cat. # 354248, BD Biosciences) at a 1:1 (v/v) ratio and injected subcutaneously into the right flank of female nude mice, as established in recent preclinical modeling protocols (Wen et al., 2022). When tumors became palpable (12 days post-injection), mice were randomized into five groups (n = 8 per group): vehicle control (saline solution, *i. g*, every other day); Low-dose MHO7 (25 mg/kg, *i. g*, every other day); Medium-dose MHO7 (50 mg/kg, *i. g*, every other day); High-dose MHO7 (100 mg/kg, *i. g*, every other day) and ADR (3 mg/kg, *i. p*, every 3 day). Tumor dimensions were measured every 2 days using digital calipers, and tumor volume was calculated as V= (length × width^2^)/2. After 15 days of treatment, mice were sacrificed and tumor, major organs (liver, kidney, spleen, heart, stomach and intestines) and blood were collected for subsequent analysis. Serum was isolated from clotted blood by centrifugation (2000×g, 10 min, 4 °C) for subsequent biochemical analyses.

### 2.9 Transcriptomic profiling

Transcriptomic profiling was conducted using the RNA sequencing approach. Total RNA was extracted from cells or tissue samples as described in 2.5. RNA integrity was assessed using the RNA Nano 6000 Assay Kit of the Bioanalyzer 2100 system, and samples with RNA integrity number values above 8.0 were selected for library preparation. Library preparation for transcriptome sequencing used total RNA as input, purifying mRNA, synthesizing cDNA, and performing PCR. Clustering was done on a cBot system and sequencing on an Illumina Novaseq platform. In data analysis, raw data was processed by fasts to obtain clean data. Reads were mapped to the reference genome using Hisat2. Novel transcripts were predicted by StringTie, and gene expression levels were quantified by featureCounts and FPKM calculation. Differential expression analysis was performed using DESeq2 or edgeR. Functional enrichment analysis, including Gene Ontology and KEGG pathway annotations, was carried out using clusterProfiler.

### 2.10 Immunohistochemistry (IHC) analysis

For immunohistochemical staining, the tissue sections were deparaffinized in xylene and rehydrated through a graded series of ethanol. Heat-induced antigen retrieval was performed by immersing the slides in 10 mM sodium citrate buffer (pH 6.0) and heating in a microwave for 15 min. After cooling to room temperature, endogenous peroxidase activity was quenched by incubating the sections with 3% hydrogen peroxide for 10 min. To block non-specific binding, the sections were incubated with 5% bovine serum albumin for 1 h at room temperature. Subsequently, the slides were incubated overnight at 4 °C with specific primary antibodies. Following three washes with PBS, the sections were incubated with the corresponding horseradish peroxidase (HRP)-conjugated secondary antibodies for 1 h at room temperature. The immunoreactive signals were visualized using DAB according to the manufacturer’s instructions. Finally, the sections were lightly counterstained with hematoxylin, dehydrated through graded ethanol, cleared in xylene, and mounted with a synthetic mounting medium.

### 2.11 Image analysis

All image analyses were performed using ImageJ. For quantitative analysis, mean fluorescence intensities were measured and background fluorescence was subtracted from a cell-free region of the same image, and the corrected fluorescence intensity was normalized to cell area. The protein expression levels in the IHC sections were semi-quantitatively evaluated using the Histological score (H-score) method (Hirsch et al., 2003). Briefly, the H-score was calculated based on both the proportion of positively stained cells and their corresponding staining intensity across multiple randomly selected high-power fields. Taining intensity was scored as 0 (negative), 1 (weak), 2 (moderate), or 3 (strong), and the percentage of cells at each intensity level was estimated.

### 2.12 Plasma biochemistry panel

Plasma biochemical analyses were conducted using an automated clinical chemistry analyzer (Beckman Coulter AU480) with commercially available reagent kits. The panel included the following analytes: serum alanine aminotransferase (ALT), aspartate aminotransferase (AST), the AST/ALT ratio, creatinine (Cr), uric acid (UA), creatine kinase (CK), CK-MB, and lactate dehydrogenase (LDH) levels.

### 2.13 Statistical analysis

Statistical analyses were performed using GraphPad Prism 8 (GraphPad Software, San Diego, CA, United States of America). Data are presented as mean ± standard deviation (SD). For comparisons between two independent groups, an unpaired two-tailed Student’s t-test was used. For comparisons among three or more groups, one-way analysis of variance (ANOVA) followed by Tukey’s *post hoc* test was applied. A value of p < 0.05 was considered statistically significant. Statistical significance is indicated in the figures as follows: *p < 0.05, **p < 0.01, ***p < 0.001; *n. s.*, not significant.

## 3 Results

### 3.1 MHO7 inhibits both adhesion to extracellular matrix components and motility of MDA-mb-231 cells

To investigate the impact of MHO7 on the migratory and invasive capacities of MDA-MB-231 cells, we conducted wound healing and Transwell assay. Quantitative analysis revealed that MHO7 treatment significantly reduced wound closure areas relative to the control (Figures 1A,B). Consistently, the invasion and migration cell counts were significantly decreased under MHO7 treatment (Figures 1D,E). Moreover, MHO7 markedly reduced the adhesion capacity of MDA-MB-231 cells, as quantified by reduced cell attachment to fibronectin-coated surfaces following MHO7 treatment (Figure 1C). Immunofluorescence analysis showed that MHO7 increased the accumulation of β-catenin at E-cadherin-positive cell–cell junctions, as indicated by the enhanced colocalization of E-cadherin and β-catenin signals (Figures 1F,G). Moreover, the mRNA expression of *CDH1* was significantly upregulated, whereas the expression of *MMP-2* and *MMP-9* was decreased under MHO7 treatment (Figures 1H,I). Collectively, these data demonstrate that MHO7 strengthens adherens junctions, thereby inhibiting ECM degradation–dependent motility in MDA-MB-231 cells.

**FIGURE 1:**
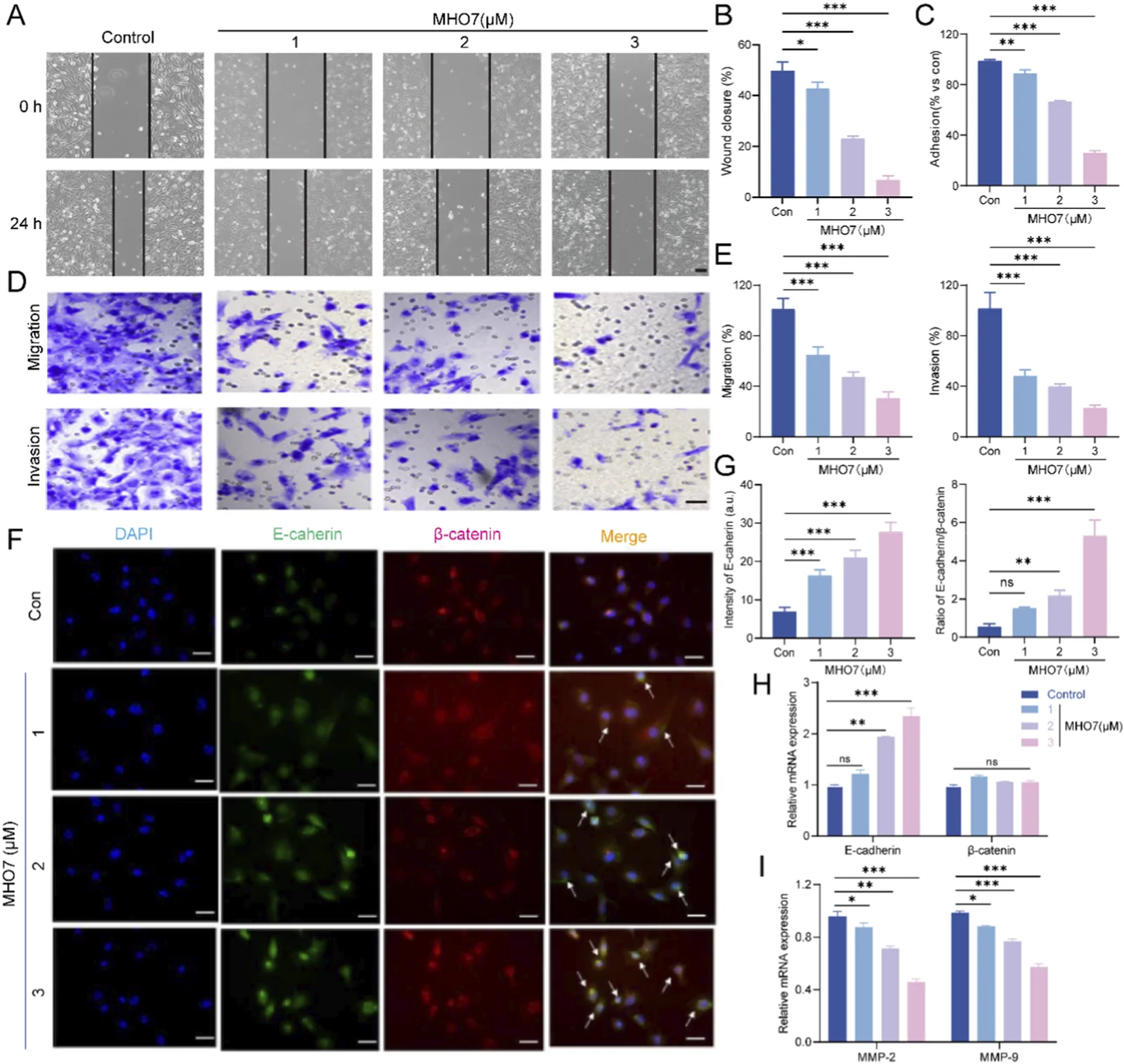
MHO7 inhibits both adhesion to extracellular matrix components and motility of MDA-MB-231 cells. **(A,B)** Representative images and quantitative analysis of the wound healing at 0 and 24 h after MHO7 treatment. Scale bar = 50 μm. **(C)** The adhesion of MDA-MB-231 cells was quantitatively assessed by measuring the percentage of cells attached to fibronectin-coated surfaces. Data are expressed as the percentage of attached cells relative to the control. **(D,E)** Representative images and quantitative analysis of cell invasion and migration under MHO7 treatment. Scale bar = 50 μm. Were assessed by Transwell assay. **(F,G)** Representative immunofluorescence images showing the expression and subcellular localization of E-cadherin and β-catenin in cells treated with increasing concentrations of MHO7. Arrows indicate regions of co-localization of E-cadherin and β-catenin at cell–cell junctions. Scale bar: 20 µm (H) RT-qPCR analysis of CDH1 and CTNNB1 mRNA expression levels (I) RT-qPCR analysis of MMP-2 and MMP-9 mRNA expression levels. n = 3. Data were presented as mean ± SD. *p < 0.05, **p < 0.01, ***p < 0.001 vs. control group, n. s.: no significance.

### 3.2 MHO7 down-regulates focal adhesion signaling in MDA-MB-231 cells

Our previous study elucidated the mechanism underlying MHO7’s anti-tumor effect, with a focus on upregulated differentially expressed genes (DEGs) enriched in ER stress-related pathways. Here, we re-analyzed transcriptome sequencing data from MHO7-treated MDA-MB-231 cells and identified 3,107 upregulated and 3,039 downregulated DEGs (|log_2_FC|> 0, padj <0.05). Subsequently, KEGG pathway enrichment analysis of the downregulated DEGs revealed significant enrichment of pathways functionally associated with focal adhesion, including focal adhesion itself, regulation of the actin cytoskeleton, and adherens junction, suggesting that attenuation of focal adhesion signaling may underlie MHO7’s anti-motility effect (Figure 2A). Consistently, GO enrichment analysis revealed significant enrichment of biological processes, cellular components, and molecular functions associated with cell-cell junctions and focal adhesion (Figure 2B). We performed a gene network analysis based on the differentially expressed genes (DEGs) enriched in the Focal Adhesion pathway, and the global network reveals that PTK2 (FAK) and SRC serve as central nodes interconnected with upstream integrin signaling components (Supplementary Figures S1A–B). These findings were further validated at the protein level. Western blot analysis revealed significant downregulation of key regulators of focal adhesion signaling, including FAK and its activated form p-FAK, as well as Src and its phosphorylated counterpart (p-Src) in MHO7-treated MDA-MB-231 cells (Figure 2C). To further investigate the impact of MHO7 on cytoskeletal dynamics, we evaluated the structural organization of filamentous actin (F-actin) using phalloidin immunofluorescence staining. As shown in Figure 2D, control MDA-MB-231 cells exhibited a typical highly invasive, spread-out morphology characterized by well-organized, prominent F-actin stress fibers traversing the cytoplasm. In contrast, MHO7 treatment induced profound morphological alterations and the disruption of the cytoskeletal architecture, indicating that MHO7 effectively compromises the structural integrity of the actin cytoskeleton. Together, these findings indicate that MHO7 downregulates focal adhesion signaling in MDA-MB-231 cells.

**FIGURE 2:**
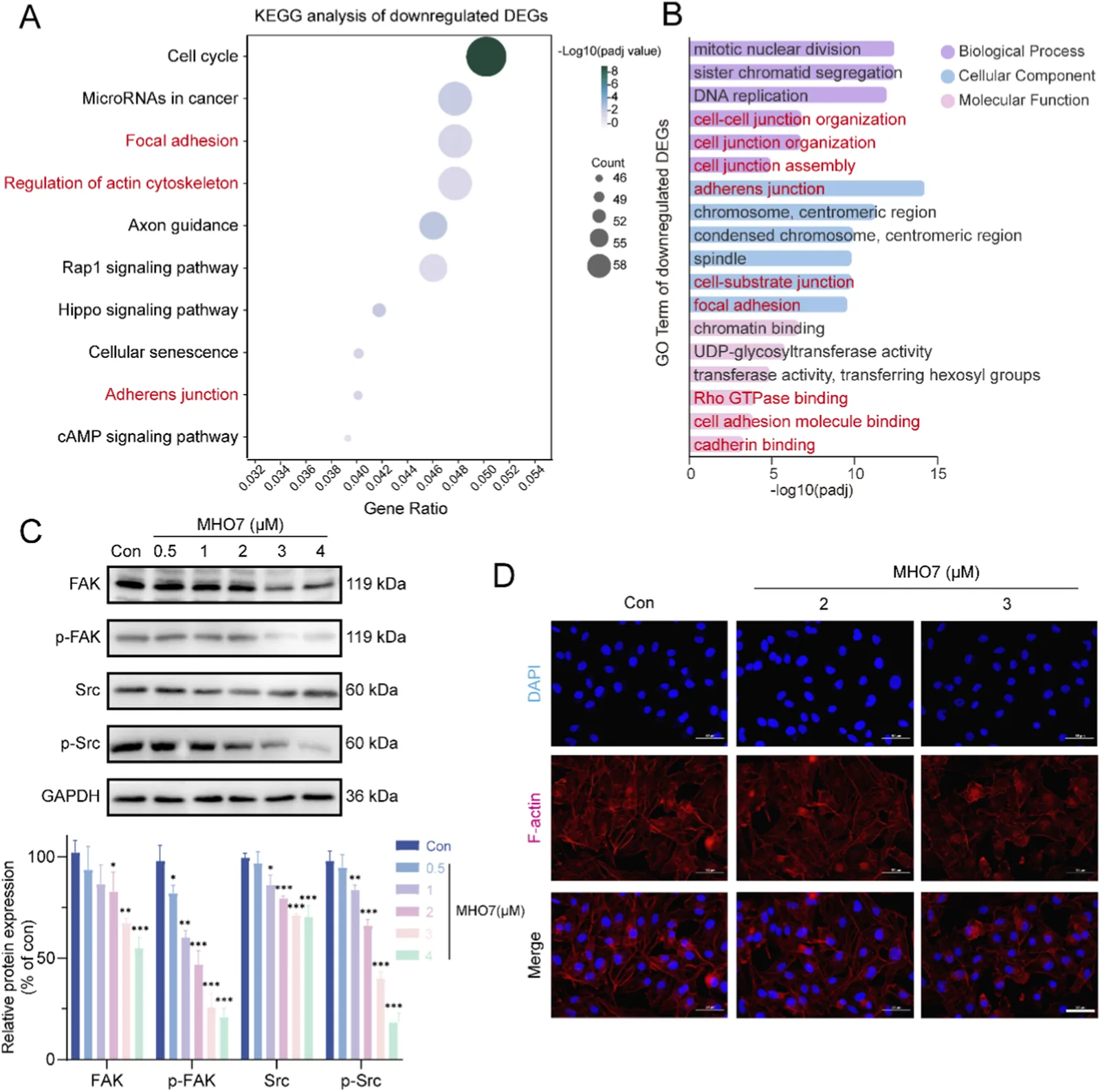
MHO7 downregulates focal adhesion signaling in MDA-MB-231 cells. **(A)** The KEGG analysis of downregulated DEGs in MDA-MB-231 cells treated with MHO7. **(B)** The GO analysis of downregulated DEGs in MDA-MB-231 cells treated with MHO7. **(C)** Western blot analysis showing the expression levels of focal adhesion associated proteins FAK, p-FAK, Src and p-Src in MDA-MB-231 cells after treatment with MHO7. n = 3. **(D)** Representative images of IF staining showing morphological alterations and structural disruption of F-actin fluorescence in MDA-MB-231 cells after MHO7 treatment. Scale bar = 50 μm. Data were presented as mean ± SD. *p < 0.05, **p < 0.01, ***p < 0.001 vs. control group.

### 3.3 MHO7 inhibits TNBC tumor growth *in vivo*

To evaluate the *in vivo* antitumor efficacy of MHO7, we established a subcutaneous TNBC xenograft model in BALB/c nude mice by subcutaneous injection of MDA-MB-231 cells. Following tumor establishment, mice were randomized into five groups and treated as follows: vehicle, MHO7 at 25, 50, or 100 mg/kg administered orally every 2 days, or doxorubicin (ADR) at 3 mg/kg administered intraperitoneally every 3 days (Figure 3A). As shown in Figures 3B,C, MHO7 administration significantly suppressed both tumor volume and growth rate at the 50 and 100 mg/kg doses, whereas no statistically significant inhibition was observed at the 25 mg/kg dose. No statistically significant difference in tumor volume was observed among the 50 mg/kg, and 100 mg/kg MHO7 treatment groups, and the 3 mg/kg ADR group. Although ADR significantly suppressed tumor growth and reduced tumor volume, it induced three treatment-related deaths in the group, indicating substantial *in vivo* toxicity. The final tumor weights were significantly lower in the 50 mg/kg MHO7 and ADR treatment groups compared to vehicle-treated mice (Figure 3D). Correspondingly, tumor growth inhibition (TGI) revealed that both the 50 mg/kg MHO7 and the 3 mg/kg ADR treatment groups exhibited robust antitumor efficacy, significantly superior to the other groups (Figure 3E). Although oral administration of MHO7 did not yield a statistically significant dose-dependent inhibition of tumor growth across all groups, histopathological analysis revealed a visual dose-dependent increase in tumor necrosis area following MHO7 treatment (Figure 3F; Supplementary Figure S2B). Given that the 50 mg/kg dose of MHO7 demonstrated the most potent numerical antitumor efficacy *in vivo*, immunohistochemical analysis was performed on tumor tissues from mice treated with this dose. Immunohistochemical analysis revealed that MHO7 treatment significantly increased cleaved caspase-3 expression and decreased CD31 expression relative to the vehicle, indicating that MHO7 promoted tumor cell apoptosis and inhibited tumor angiogenesis (Figures 3G–J). Notably, the significant downregulation of p-FAK and p-Src expression in tumor tissues indicated that MHO7 effectively suppresses focal adhesion signaling *in vivo* (Figures 3K–N). This finding is consistent with the mechanism of action observed at the cellular level and collectively supports MHO7’s antitumor efficacy.

**FIGURE 3:**
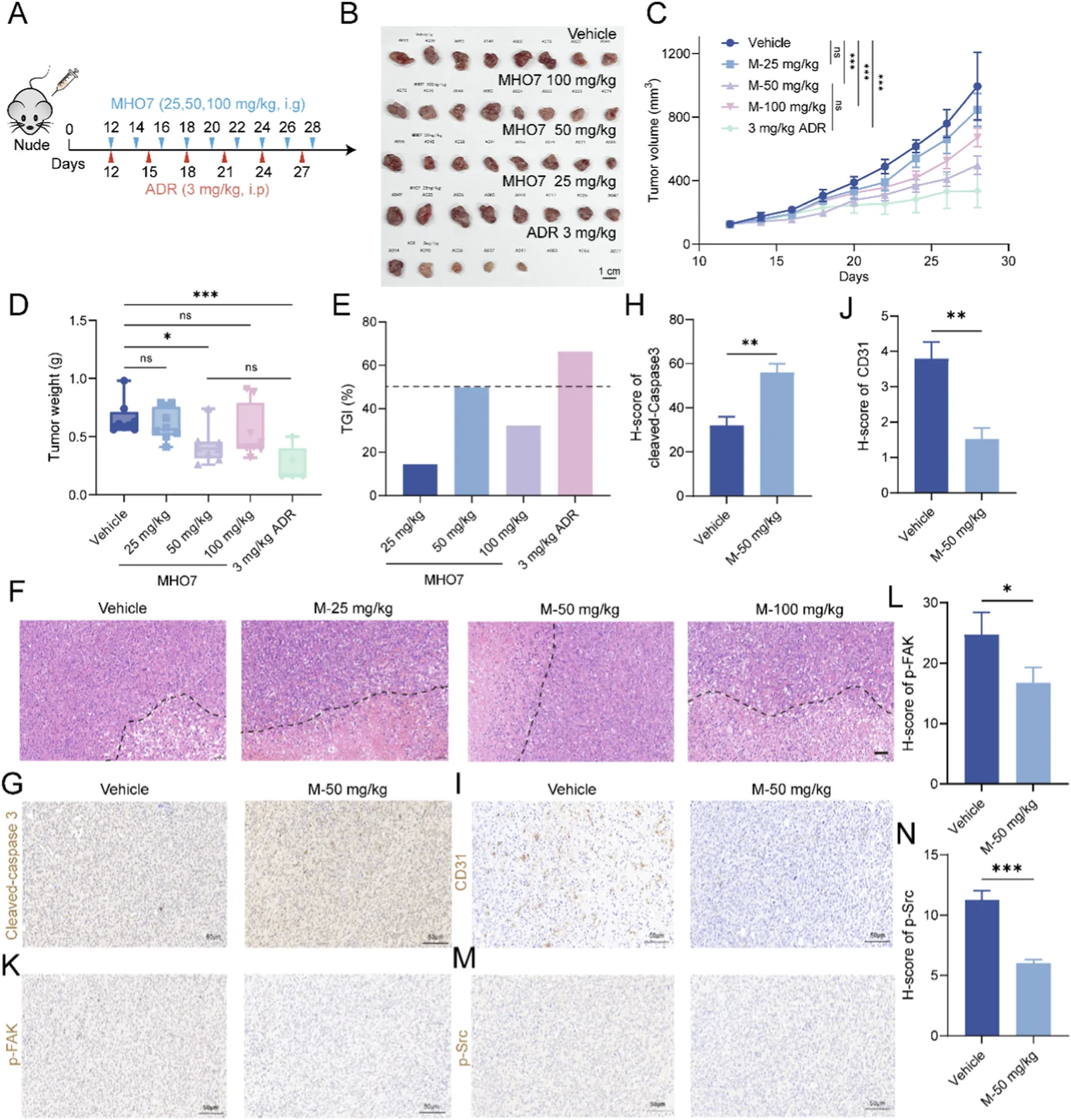
MHO7 suppresses TNBC tumor growth *in vivo*. **(A)** Schematic illustration of the *in vivo* experimental protocol (n = 6 mice/group). **(B)** Representative photographs of subcutaneous breast tumors harvested from mice. **(C)** Tumor growth curves monitored every 2 days during the treatment period. **(D)** Final tumor weights measured at experimental termination. **(E)** Tumor growth inhibition (TGI) calculated from final tumor weights. **(F)** H&E staining of tumor tissues from the MHO7-treated group. The necrotic regions were demarcated using dashed lines. Scale bar = 50 μm. **(G–J)** Representative immunohistochemical staining images and quantitative analysis of cleaved-caspase-3 and CD31 in tumor tissues from vehicle- or MHO7 (50 mg/kg)-treated mice. **(K–N)** Representative immunohistochemical staining images and quantitative analysis of p-FAK and p-Src in tumor tissues from vehicle- or MHO7 (50 mg/kg)-treated mice. Scale bar = 50 μm. Data for tumor volumes are presented as mean ± SEM, while other quantitative data are presented as mean ± SD. *p < 0.05, **p < 0.01, ***p < 0.001, n. s.: no significance.

### 3.4 MHO7 suppresses key downstream effectors of the wnt and PI3K signaling pathways

To determine whether the *in vivo* mechanism of MHO7 aligns with its cellular-level effects, transcriptome profiling was performed on tumor tissues from vehicle- or MHO7 (50 mg/kg)-treated mice. Volcano plot analysis identified 1,135 upregulated and 1,090 downregulated DEGs (|log_2_FC|>0, padj< 0.05) (Figure 4A). Consistent with the pathway enrichment results observed in MHO7-treated MDA-MB-231 cells, KEGG and GO analyses of tumor-derived DEGs revealed focal adhesion as a significantly enriched signaling regulated by MHO7 (Figures 4B,C). Notably, the Wnt signaling pathway, the canonical downstream effector of focal adhesion signaling, was also robustly downregulated in both analyses. To functionally validate this transcriptional signature, we assessed key components of the canonical Wnt/β-catenin pathway by Western blot. As shown in Figure 4D, MHO7 treatment induced a dose-dependent decrease in nuclear and cytoplasmic β-catenin levels, reflecting the i enhanced proteasomal degradation of cytoplasmic β-catenin and the consequent blockade of its nuclear translocation. Moreover, MHO7 upregulated GSK-3β and E-cadherin protein expression while downregulating total β-catenin levels, collectively supporting the suppression of canonical Wnt/β-catenin signaling (Figure 4E). Concurrently, Western blot analysis demonstrated reductions in the phosphorylation levels of PI3K (p-PI3K) and Akt, confirming the inhibition of PI3K/Akt signaling activity (Figure 4F). Immunofluorescence staining further revealed disrupted Paxillin localization and severe disorganization of the F-actin structural network in tumor tissues upon MHO7 treatment, indicating impaired FAK-mediated adhesion complex assembly *in vivo* (Figure 4G). Consistent with these findings, immunohistochemical analysis confirmed significant downregulation of p-PI3K and p-Akt in MHO7-treated tumor sections (Figure 4H). Collectively, these findings indicate that MHO7 suppresses key downstream effectors of the Wnt and PI3K signaling pathways, both of which are interconnected with focal adhesion signaling in MDA-MB-231 cells.

**FIGURE 4:**
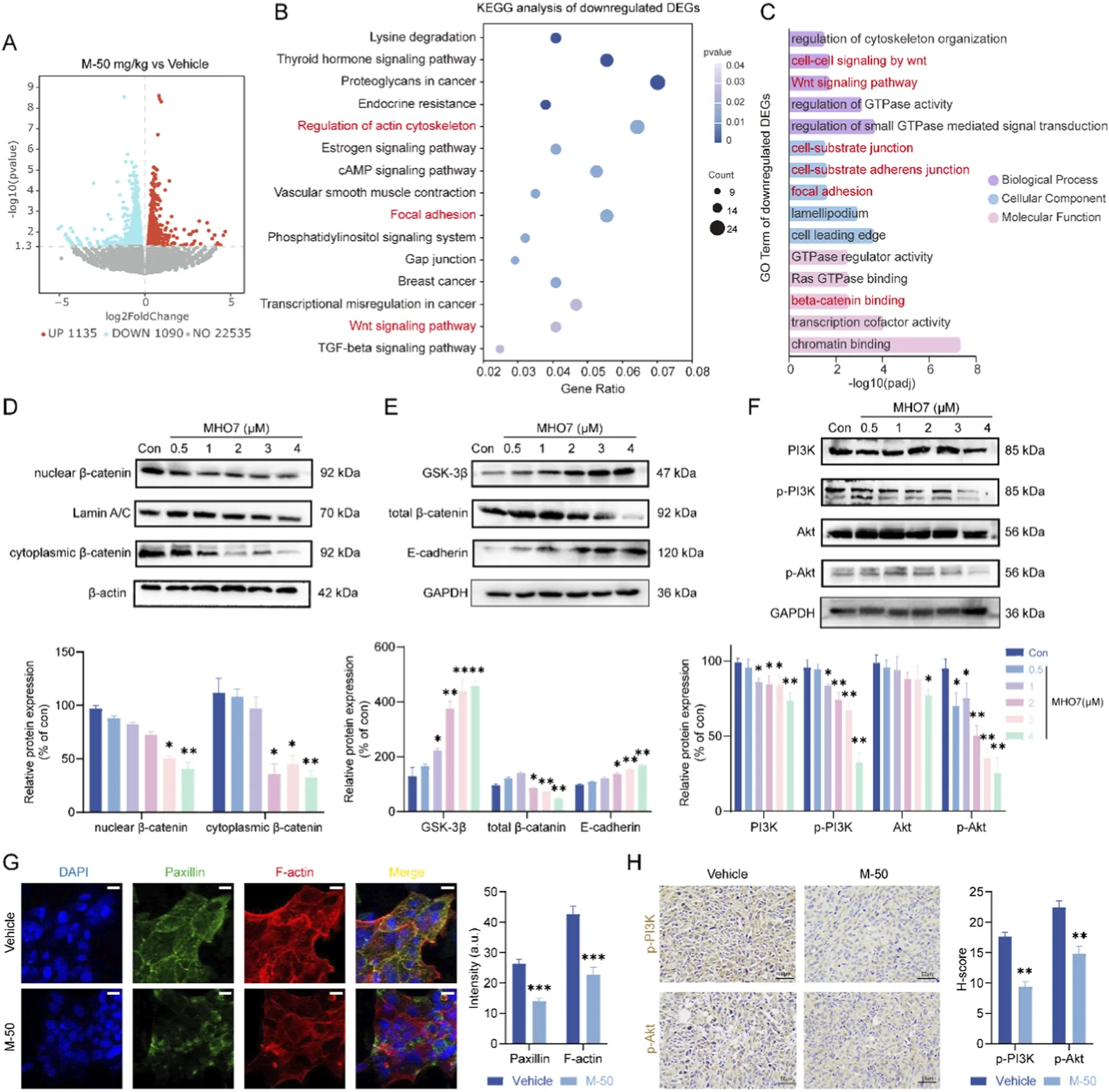
MHO7 suppresses key downstream effectors of the Wnt and PI3K signaling pathways. **(A)** Volcano plot showing differentially expressed genes (DEGs) following MHO7 treatment. **(B)** KEGG enrichment analysis of downregulated DEGs in tumor tissues treated with MHO7 (50 mg/kg). **(C)** GO enrichment analysis of downregulated DEGs in tumor tissues treated with MHO7 (50 mg/kg). **(D,E)** Western blot analysis showing the expression levels of Wnt signaling-associated proteins β-catenin, GSK-3β and E-cadherin and cytoplasmic β-catenin in MDA-MB-231 cells with MHO7 treatment. n = 3. **(F)** Western blot analysis showing the expression levels of PI3K/Akt signaling-associated proteins PI3K, p-PI3K, Akt and p-Akt in MDA-MB-231 cells with MHO7 treatment. n = 3. **(G)** Representative images of IF staining showing disrupted paxillin localization and structurally dismantled F-actin in tumor tissue after MHO7 treatment. Scale bar = 50 μm. **(H)** Representative immunohistochemical staining images and quantitative analysis of p-PI3K and p-Akt in tumor tissues from vehicle- or MHO7 (50 mg/kg)-treated mice. Scale bar = 50 μm. Data were presented as mean ± SD. *p < 0.05, **p < 0.01, ***p < 0.001.

### 3.5 MHO7 showed no significant organ toxicity *in vivo*

To assess systemic toxicity, histopathological analyses of major organs and serum biochemical parameters were evaluated. As shown in Figure 5A, histopathological analyses of major organs (heart, liver, kidney, and spleen) revealed no overt structural abnormalities or pathological lesions following treatment with various doses of MHO7. In contrast, mice treated with ADR exhibited severe myocardial cell deformation, marked hepatic congestion, and nuclear pyknosis with chromatin condensation in renal and splenic cells, collectively indicating that ADR induces significant organ toxicity and markedly stronger adverse effects compared with MHO7. Moreover, while mice in the vehicle- and MHO7-treated group exhibited a steady increase in body weight throughout the intervention period, mice in the ADR group showed progressive body weight loss, suggesting that ADR induced significantly greater systemic toxicity than MHO7 (Figure 5B). We further evaluated gastrointestinal toxicity following oral administration of MHO7 through histopathological analysis, and results revealed no significant pathological alterations in the gastric or intestinal tissues of mice compared with those in the vehicle group (Figure 5C). No significant differences were observed in serum alanine aminotransferase (ALT), aspartate aminotransferase (AST), the AST/ALT ratio, creatinine (Cr), uric acid (UA), creatine kinase (CK), CK-MB, or lactate dehydrogenase (LDH) levels following MHO7 treatment, indicating no evidence of hepatotoxicity, nephrotoxicity, or cardiotoxicity (Figures 5D–K). However, Cr, UA, CK, and CK-MB levels were significantly altered, whereas LDL cholesterol levels were elevated relative to the vehicle control, suggesting potential nephrotoxicity and cardiotoxicity induced by ADR in mice. These findings indicate that MHO7 was well tolerated in mice, with no evidence of systemic, hepatic, renal, or cardiac toxicity, supporting its translational potential as a therapeutic candidate.

**FIGURE 5:**
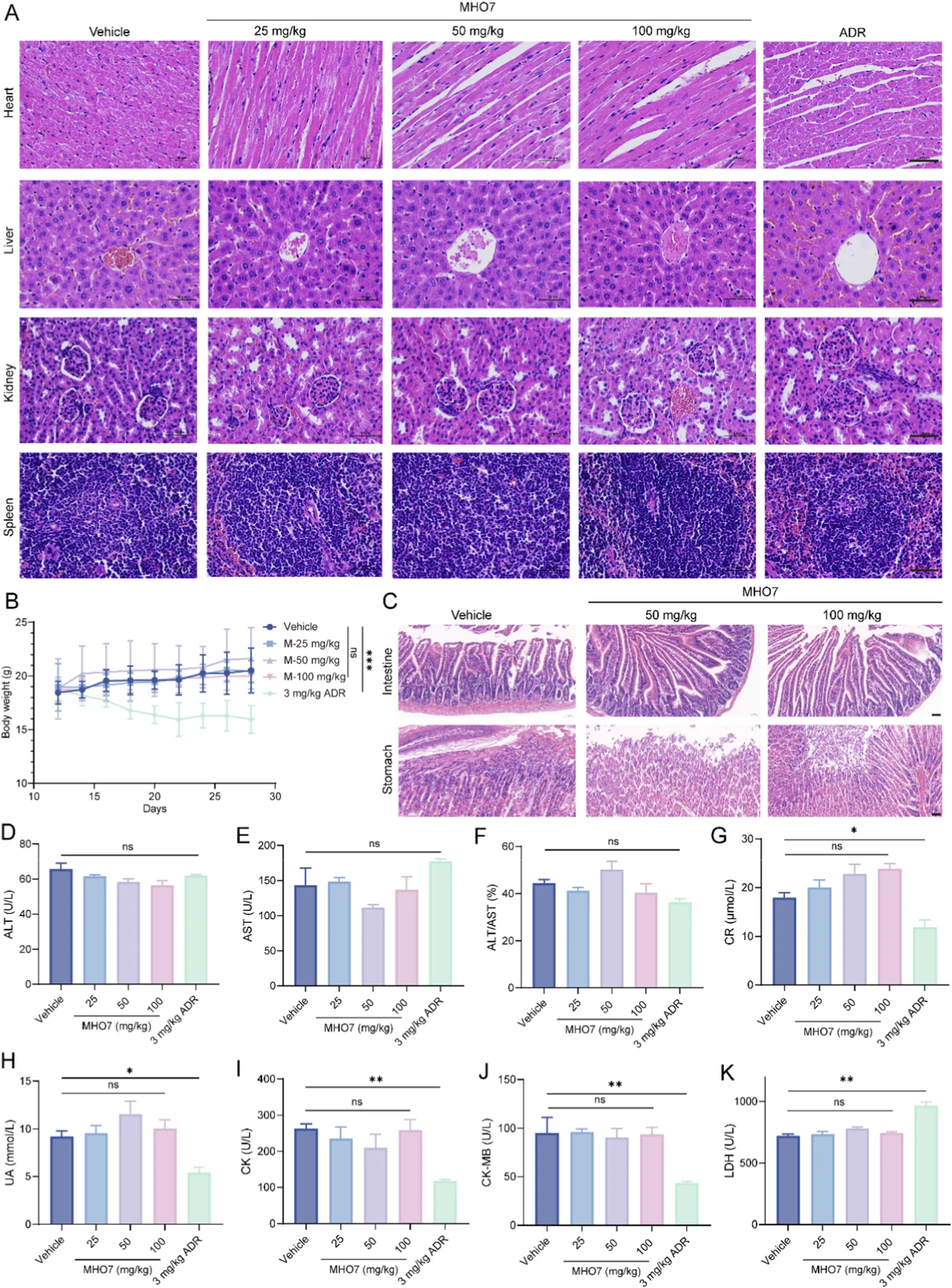
MHO7 showed no significant organ toxicity *in vivo*. **(A)** Representative H&E staining images of major organs (heart, liver, kidney, and spleen) collected from mice. Scale bar = 50 µm. **(B)** Changes of body weight during the treatment period. **(C)** Representative H&E staining images of the gastric and intestinal tissues. Scale bar = 50 µm. Serum **(D)** ALT, **(E)** AST, **(F)** the AST/ALT ratio, **(G)** Cr, **(H)** UA, **(I)** CK, **(J)** CK-MB, or **(K)** LDH levels. n = 6 mice/group. Data were presented as mean ± SD. *p < 0.05, **p < 0.01, ***p < 0.001, n. s.: no significance.

## 4 Discussion

Although the therapeutic landscape of TNBC has expanded beyond conventional chemotherapy to include immune checkpoint inhibitors, ADCs, and PARP inhibitors, the majority of patients still fail to achieve sustained clinical benefit. This underscores the urgent need for novel, highly effective therapeutic agents. In this study, we demonstrated that MHO7, a novel sesterterpenoid isolated from a marine fungus, profoundly inhibited the migration and invasion of MDA-MB-231 cells. Mechanistically, we elucidated that these effects are associated with the disruption of focal adhesion signaling and the profound attenuation of FAK activation. Furthermore, MHO7 exhibited robust antitumor efficacy *in vivo* subcutaneous TNBC mouse models without inducing overt systemic toxicity.

Owing to their structurally diverse chemical scaffolds and inherent multi-target pharmacological profiles, natural compounds often confer distinct therapeutic advantages over single-target agents in managing highly heterogeneous malignancies like TNBC. Consistent with this, ophiobolins, a unique class of fungal sesterterpenoids, exhibit potent, multi-pathway antitumor activities (Aebisher et al., 2026; Ranganathan et al., 2026). Following our successful isolation of MHO7 from the mangrove fungus *Aspergillus ustus 094,102* (Chai et al., 2016), we have systematically investigated its pharmacological potential. We previously established that MHO7 functioned as an estrogen receptor alpha (ERα) degrader in ER-positive breast cancer (Zhao et al., 2019), and effectively halted TNBC growth by inducing immunogenic cell death (Wen et al., 2022). Expanding upon these foundational findings, the current study elucidated the anti-tumor dimensions of MHO7 in TNBC. Given that high invasiveness is a hallmark of TNBC, our findings that MHO7 not only curtails cellular motility but also actively fortifies adherens junctions provides critical insights into its therapeutic promise. Importantly, considering the utilized *in vitro* concentrations are near the sub-toxic range, this restriction of malignant spread likely reflects a combined outcome of impaired cellular motility and reduced cell viability.

Hyperactivation of the FAK signaling, a primary orchestrator of epithelial-mesenchymal transition (EMT), migration, and invasion, is widely recognized as a critical driver of the metastatic cascade and chemoresistance (Dawson et al., 2021; Sun et al., 2023; Yang et al., 2024). Consistent with this paradigm, our transcriptomic profiling of both MDA-MB-231 cells and tumor tissues revealed a significant enrichment in signatures associated with focal adhesion and cell motility. Conversely, MHO7 treatment profoundly downregulated gene sets linked to FAK regulation, including cell-cell junctions, focal adhesions, and Rho GTPase binding, strongly suggesting that MHO7 acts as a robust suppressor of the FAK signaling axis. Mechanistically, the autophosphorylation of FAK at tyrosine 397 (Y397) is the indispensable initiating event for its full activation, prompting the subsequent recruitment and activation of Src family kinases (Chen et al., 2026). Our *in vitro* and *in vivo* data clearly demonstrated that MHO7 treatment abrogated the levels of both p-FAK (Y397) and p-Src (Y419). Functionally, this MHO7-mediated FAK suppression disrupted cytoskeletal dynamics, evidenced by altered F-actin architectural organization and impaired paxillin localization, triggered apoptosis (elevated cleaved caspase-3), and curtailed angiogenesis (decreased CD31 expression). Given that the PI3K/AKT cascade is a major downstream effector of FAK, the concurrent downregulation of p-PI3K and p-AKT further corroborates the robust inhibition of FAK signaling by MHO7, highlighting the broader significance of targeting the highly conserved PI3K/AKT network with natural products across distinct cancer types (Zhou et al., 2025).

A major hurdle in targeting FAK lies in its extensive crosstalk with other oncogenic networks, notably the canonical Wnt/β-catenin pathway, which coregulates cytoskeletal reorganization and cell motility (Worthmuller and Ruegg, 2020; Schlaepfer et al., 2024). According to its dependence on the key mediated β-catenin, Wnt signaling pathway is divided into the β-catenin-dependent canonical and independent non-canonical pathways (Liu et al., 2022). The canonical branch characterized by the accumulation and nuclear translocation of β-catenin protein, regulates key cellular processes, including cytoskeletal reorganization, cell motility, and stemness maintenance, and engages in bidirectional functional crosstalk with FAK (Katoh, 2024; Schlaepfer et al., 2024). Consequently, dual suppression of the FAK-Wnt signaling axis has emerged as a compelling therapeutic strategy. The increased co-localization of E-cadherin and β-catenin at cell–cell junctions suggests that MHO7 may promote the stabilization of adherens junctions and reinforce epithelial cell–cell adhesion. In parallel, the reduced nuclear-to-cytoplasmic ratio of β-catenin indicates decreased nuclear accumulation of β-catenin, which may contribute to the suppression of β-catenin-dependent transcriptional programs associated with tumor-cell migration and invasion. Strikingly, our transcriptomic analysis revealed a significant suppression of the Wnt signaling pathway following MHO7 treatment in TNBC mice models. Subsequent experimental validation confirmed that MHO7 dose-dependently reduced the expression levels of nuclear β-catenin and GSK-3β. These findings imply that MHO7’s potent anti-TNBC efficacy may be partially driven by a dual inhibitory effect on the FAK-Wnt crosstalk, thereby offering a novel therapeutic mechanism that could complement existing chemo- or immunotherapies.

Interestingly, while MHO7 exhibited a dose-dependent inhibitory effect on MDA-MB-231 cell migration and invasion *in vitro*, our *in vivo* therapeutic evaluation via oral administration revealed a non-monotonic dose-response trend, where the intermediate dose (50 mg/kg) demonstrated the optimal numerical anti-tumor efficacy. While optimal immunotherapy responses or tumor vascular normalization at intermediate doses may theoretically contribute to this phenomenon within the complex tumor microenvironment (Glasmacher et al., 2025; Kabir et al., 2026), we must acknowledge that this bell-shaped response could largely reflect experimental variability due to the limited sample size. Furthermore, from a pharmacokinetic perspective, high doses of natural sesterterpenoids often encounter challenges regarding gastrointestinal absorption saturation or drug aggregation *in vivo* upon oral administration, potentially limiting the proportional increase in bioavailability (Atriya et al., 2023). Therefore, our findings underscore the critical importance of optimizing the dosing regimen and administration route of MHO7 in future studies.

Despite these findings, several limitations warrant acknowledgment. First, while MHO7 treatment profoundly attenuated FAK/Src signaling, the present data do not establish FAK as a direct molecular target. The observed pathway suppression might be an indirect consequence of MHO7 interacting with an unidentified upstream regulator. Second, to establish strict causality rather than mere correlation between FAK attenuation and the observed phenotypes, future rescue experiments are required. Although preliminary systemic toxicity and pharmacokinetics were evaluated here and in our previous report (Tian et al., 2019), these data rely entirely on preclinical models, and future validation in patient-derived xenografts or clinical samples is imperative for translation. Third, given the intrinsic molecular heterogeneity of TNBC, relying solely on a single mesenchymal-like cell line (MDA-MB-231) for our *in vitro* assays limits the broad applicability of our findings. Therefore, our mechanistic conclusions regarding MHO7-mediated pathway suppression should be interpreted as specific to this cell context. Our subsequent phase of research will focus on precisely mapping MHO7’s direct binding targets, conducting comprehensive pharmacokinetic profiling, and extending evaluations across patient-derived TNBC models, followed by the structural optimization of this compound to solidify its foundation for clinical application (Li et al., 2026).

In conclusion, our study identifies MHO7, a marine-derived sesterterpenoid, as a potent suppressor of TNBC tumor growth and cell motility. This anti-tumor efficacy is mechanistically associated with the robust attenuation of the FAK signaling pathway and its oncogenic crosstalk networks. These findings underscore the translational potential of MHO7 as a promising lead compound for the treatment of highly aggressive TNBC.

## Data Availability

The differentially expressed genes (DEGs) identified from RNA-seq analysis, together with all other data underlying the conclusions of this study, are provided in the main text and its associated Supplementary Files. Requests for additional information or materials should be addressed to the corresponding author.
