# Supplementary material for: A marine-derived sesterterpenoid inhibits migration and invasion of triple-negative breast cancer cells by suppressing the FAK signaling pathway
Source: Front Cell Dev Biol. 2026 Oct 15;14:1967923. doi: 10.3389/fcell.2026.1967923 (PMC13716777; doi:10.3389/fcell.2026.1967923)
Supplement: Supplementary file 1 [file Image1.pdf]

Supplementary Figures

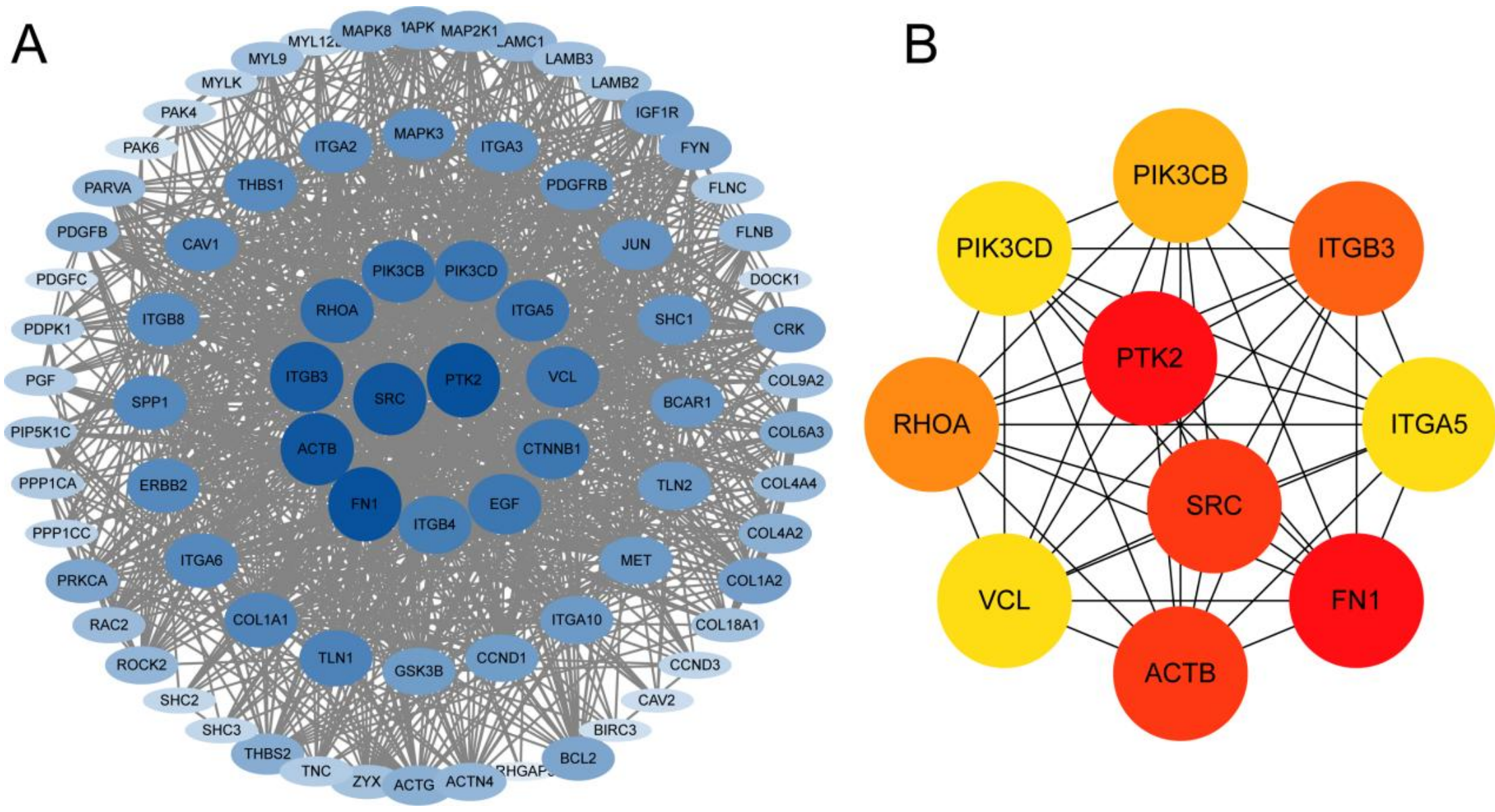

Supplementary Figure S1 Gene interaction network analysis of DEGs enriched in focal adhesion pathway upon MHO7 treatment. (A) Protein–protein interaction (PPI) network constructed from differentially expressed genes (DEGs) associated with focal adhesion pathway in MHO7-treated MDA-MB-231 cells compared with vehicle control. (B) Top 10 hub genes ranked by degree value. The color gradient from yellow to red represents increasing degree values, with PTK2, SRC, and FN1 showing the highest degree.

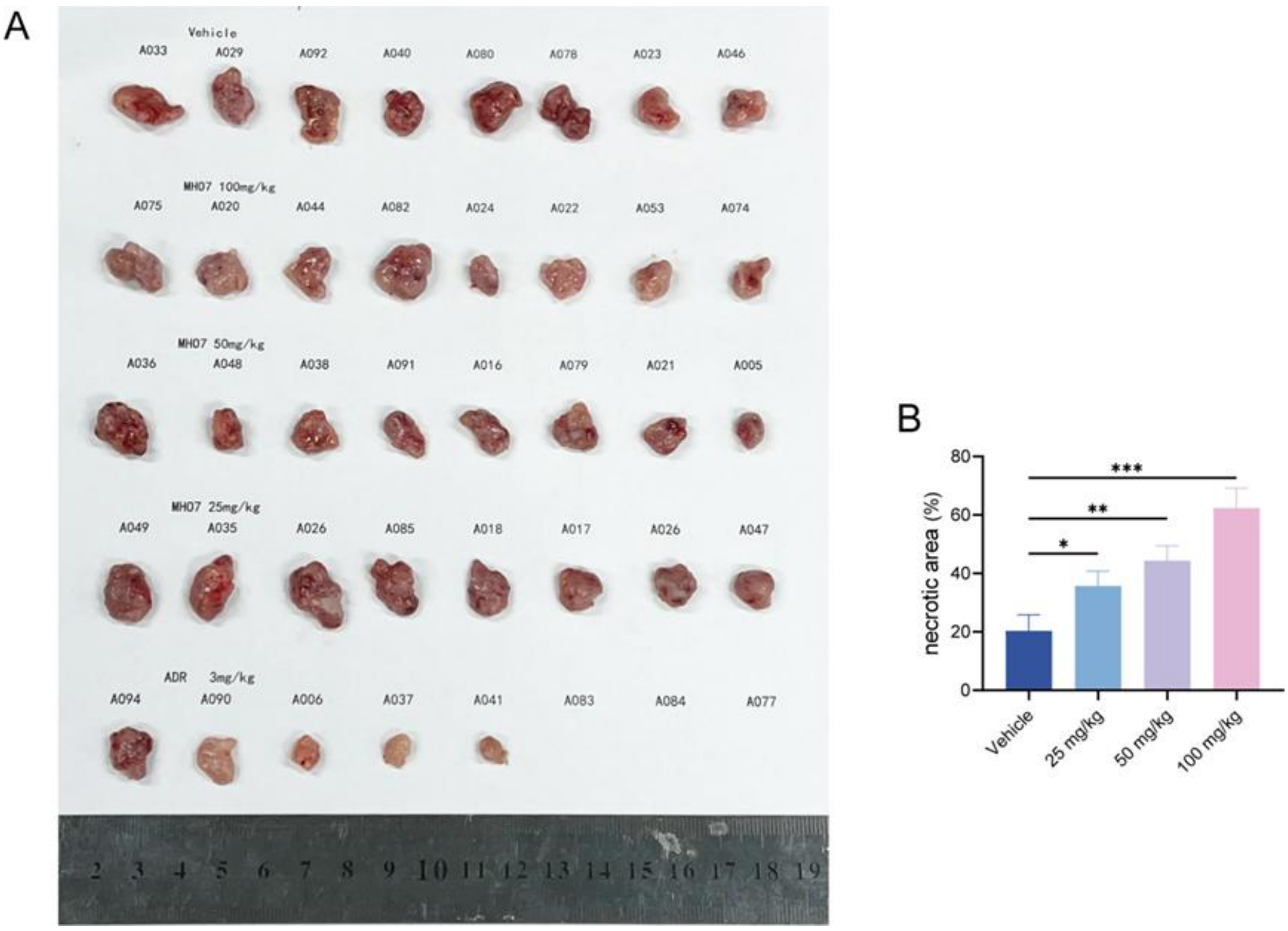

Supplementary Figure S2. (A) Enlarged and high-resolution version of the images shown in Figure 3B. The mouse identification numbers and experimental group assignments are provided to facilitate clear matching between individual mice and their corresponding tumor samples. (B) The necrotic area percentage across treatment groups. $^{*}p < 0.05$, $^{**}p < 0.01$, $^{***}p < 0.001$ vs. vehicle group.
